# The impact of individual health on relative poverty: evidence from China

**DOI:** 10.3389/fpubh.2025.1606778

**Published:** 2025-08-05

**Authors:** Ying Liu, Gongjing Gao, Yating Sun

**Affiliations:** ^1^School of Sociology and Political Science, Anhui University, Hefei, China; ^2^School of Political Science and Law, University of Jinan, Jinan, China

**Keywords:** individual health, employment status, medical expenditures, relative poverty, human capital

## Abstract

**Background:**

Following China’s historic achievement in eradicating absolute poverty, the focus of anti-poverty policies has shifted to alleviating relative poverty, with health status playing a pivotal role in sustainable poverty reduction.

**Methods:**

Based on the 2021 Chinese General Social Survey (CGSS 2021), we employed structural equation modeling (SEM) to analyze the relationship between individual health and relative poverty.

**Results:**

The analyses showed a significant negative correlation between individual health and relative poverty. This effect occurs in two ways: by increasing employment participation and by reducing the burden of health care expenditures. It is worth noting that health-induced income loss has a greater impact on relative poverty than direct medical costs.

**Conclusion:**

These findings demonstrate that health plays a dual role in poverty governance: enhancing human capital while mitigating economic risks. This provides empirical evidence for alleviating relative poverty and advancing common prosperity through improving individual health outcomes.

## Highlights

- In the post-poverty era, China’s poverty governance has transitioned from eradicating absolute poverty to alleviating relative poverty, where individual health status plays a critical role in sustainable development.
- Improved health reduces the probability of relative poverty and provides empirical evidence for precision health interventions in poverty reduction strategies.
- Structural equation modeling (SEM) identified dual mediation mechanisms: enhanced employment participation and reduced medical expenditure burden.
- Health-induced income loss exerted a stronger influence on relative poverty than direct medical costs.

## 1 Introduction

In 2020, China achieved a comprehensive victory in its poverty eradication campaign, historically resolving absolute poverty according to national standards. However, this milestone does not signify the end of poverty-related challenges. Relative poverty is poised to grow more pronounced and persist long-term, necessitating the exploration of sustainable mechanisms to alleviate it in the post-poverty era (1, 2). Establishing such mechanisms demands a focus on endogenous development, where human capital is a critical driver (3). Within this framework, health capital has garnered significant attention from the government and academia in recent years (4–7). For China, this focus has a profoundly practical context: Disease is a major contributor to poverty in China, accounting for 44% of the poor. Coupled with the continuing impact of the COVID-19 epidemic, health-related poverty risks have become a major challenge in the post-poverty eradication era that urgently and systematically needs to be addressed (4).

Health and poverty exhibit a demonstrable correlation (3, 8, 9). Previous literature attributes the impact of individual health on poverty to two primary mechanisms. First, declining health status impairs human capital development and reduces income-generating capacity (10–12). Specifically, individuals with compromised health often face employment difficulties, limiting income access and thereby increasing poverty risk. Second, diminished individual health levels impose medical expenditure burdens (13–16), exposing individuals to poverty vulnerability. More critically, impoverished populations frequently encounter barriers to accessing timely medical interventions, exacerbating the vicious cycle of the “health-poverty trap” (17). Substantial evidence indicates a significant negative correlation between health status and relative poverty risk (6, 8, 18–21); however, relevant findings suggest this relationship is context-dependent (7).

The existing literature has provided important references for this study. However, the references still have the following limitations. First, current empirical findings on the association between individual health and relative poverty show inconsistent results. Furthermore, existing studies predominantly rely on region-specific samples rather than nationally representative data. Given China’s substantial regional heterogeneity, these geographically constrained findings may not fully capture broader patterns, raising concerns about generalizability. Second, scholarly discussions have predominantly centered on absolute poverty in both domestic and international contexts. In contrast, research on relative poverty remains comparatively underdeveloped, with its underlying mechanisms requiring further empirical investigation to clarify operational pathways.

To address these research gaps, this study establishes three objectives: First, to clarify the relationship between health and relative poverty by examining whether individual health affects relative poverty and quantifying the extent of this association. Second, to identify the mediating mechanisms through which individual health influences relative poverty formation, systematically analyzing the pathways by which health impacts poverty outcomes. Third, to conduct heterogeneity analyses considering China’s urban–rural disparities, thereby distinguishing differential health-poverty dynamics across populations. Based on these objectives, this research utilizes nationally representative data from the 2021 China General Social Survey (CGSS) and employs structural equation modeling (SEM) to empirically investigate the relationship between individual health and relative poverty. The findings provide actionable insights for developing targeted policy frameworks that address relative poverty through health-centered interventions.

## 2 Methods

### 2.1 Structural equation modeling

This study employs structural equation modeling (SEM) to examine the relationships between observed indicators and latent constructs while investigating the interrelationships among latent variables. The model specifically elucidates the pathway mechanisms through which individual health influences relative poverty status. SEM is selected for its capacity to integrate confirmatory factor analysis with path modeling, thereby enabling simultaneous estimation of multidimensional health impacts that operate as both latent determinants and mediating mechanisms, while rigorously controlling for measurement errors.

**Figure 1:**
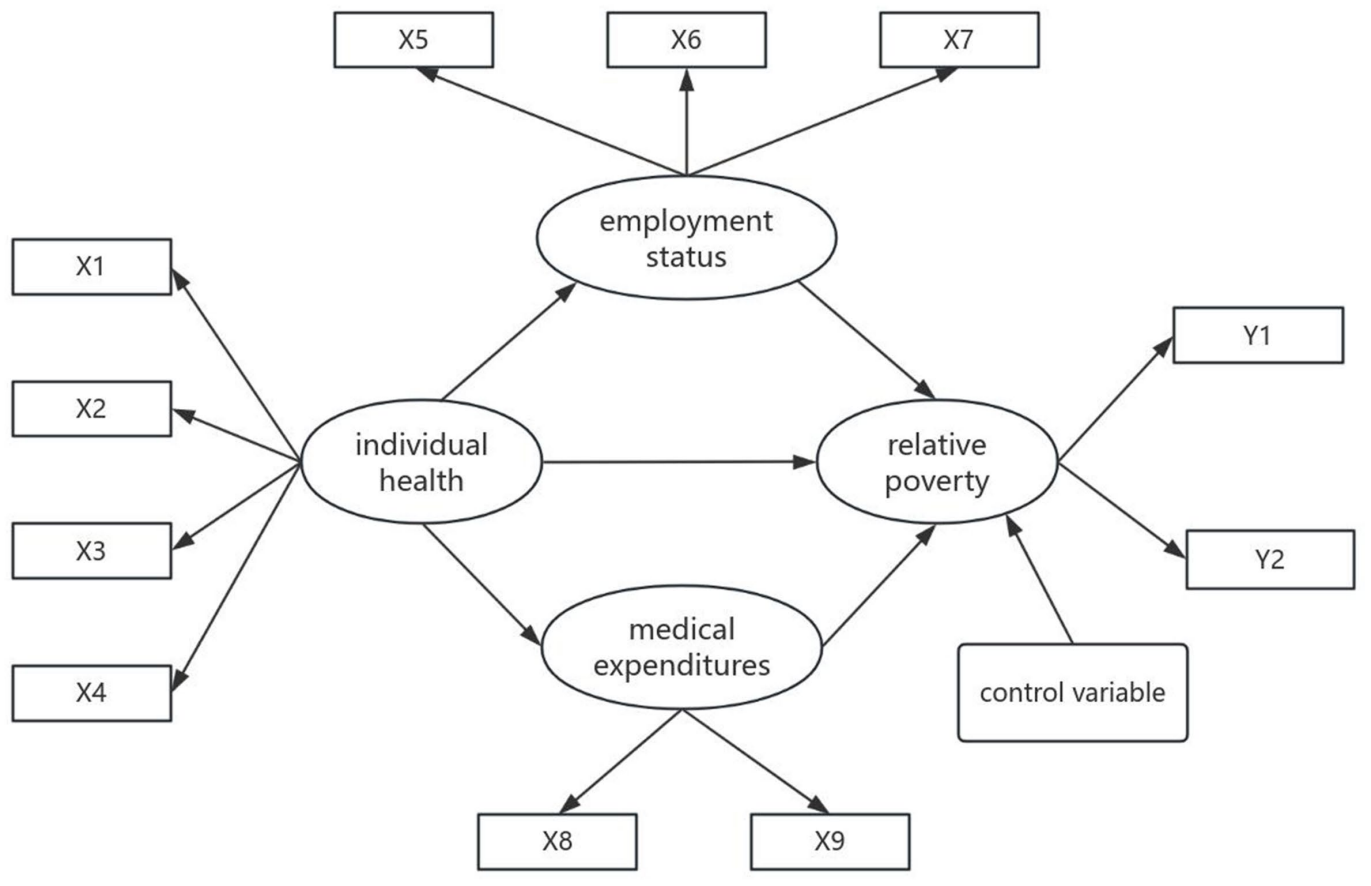
Impact pathways model of individual health on relative poverty.

Existing research provides a foundation for positing three primary mechanisms linking individual health to relative poverty. First, health exerts a direct effect on poverty, wherein enhanced health status corresponds to a reduced probability of poverty incidence. Second, health indirectly shapes poverty outcomes through employment status: healthier individuals demonstrate a greater likelihood of securing formal employment with wage benefits, which mitigates poverty risks through sustained income streams. Third, health status affects poverty via out-of-pocket medical expenditures. Such expenditures frequently trigger health shock-induced impoverishment or chronic poverty traps. The conceptual framework synthesizing these pathways is presented in Figure 1.

### 2.2 Data

The data for this study are derived from the 2021 China General Social Survey (CGSS), a comprehensive nationwide survey with repeated cross-sectional waves administered by the National Survey Research Center (NSRC) at Renmin University of China. This survey systematically collects multi-level data encompassing society, communities, families, and individuals, holding significant value for addressing diverse scientific and practical issues. This paper specifically employs the 2021 CGSS data rather than longitudinal datasets for two primary reasons: First, building upon existing research frameworks, the operationalization of key variables in this study aligns most effectively with the indicators available in the 2021 dataset. Second, given the contextual relevance of China’s landmark achievement of eradicating absolute poverty in 2020, the 2021 data are particularly suited for investigating the impact of individual health on relative poverty within the post-poverty-alleviation era. It should be noted that the 2021 data may not fully capture subsequent developments, such as the effects of the post-pandemic period and shifts in welfare policies. Nevertheless, this specific temporal window offers unparalleled insights into the initial phase of the dynamics of relative poverty following the elimination of absolute poverty. Therefore, we ultimately selected the 2021 CGSS dataset to analyze the relationship between individual health and relative poverty.

To investigate the impact of individual health on relative poverty and explore the pathways through which health influences relative poverty, we implemented the following data processing procedures. First, we employed multiple imputation methods to address missing values. This approach maximizes data completeness while ensuring compatibility with structural equation modeling requirements. After multiple imputations, we obtained five data subsets and selected the optimal subset based on the principles of rationality and optimality. Subsequently, we excluded observations with persistent missing values post-imputation to enhance analytical robustness. Through these two processing steps, we ultimately obtained 8,125 analytical samples. These samples demonstrate broad representativeness and can accurately reflect the actual conditions of Chinese society, making their use in this study both methodologically sound and empirically valid.

### 2.3 Dependent variable

Relative poverty constitutes the core dependent variable in this study. Current academic approaches to measuring relative poverty predominantly follow two paradigms: unidimensional and multidimensional frameworks. While unidimensional methods focus exclusively on income/wealth metrics, multidimensional indices incorporate supplementary dimensions such as education, health, and living standards. Given that multidimensional measurement inherently embeds health indicators, thereby preventing their isolation from the multidimensional poverty, and considering the greater operational feasibility and broader empirical comparability of unidimensional approaches, this study adopts the internationally standardized unidimensional method: the income-to-median ratio approach. Nevertheless, acknowledging that relative poverty transcends purely objective economic conditions by encompassing subjective deprivation perceptions, our operationalization integrates both objective and subjective indicators.

To achieve operationalization of relative poverty through objective indicators, an income threshold set at 50% of the median income was adopted, aligning with the OECD’s comparative poverty standard. For subjective measurement, drawing on Rojas’ (22) concept of economic deprivation perception, we employed the following approach: Respondents who “consider themselves far below average” were coded as 1 (indicating relative poverty status), while others were coded as 0. Crucially, chi-square test results revealed a statistically significant association between objective poverty indicators and subjective poverty perception (*p* < 0.001). This finding strongly supports the complementary nature of both measurement approaches in characterizing relative poverty, consequently validating the rationale for their integrated analysis.

### 2.4 Independent variables

Individual health constitutes the core explanatory variable in this study, anchored in the World Health Organization’s (WHO) 1948 definition that conceptualizes health as “a state of complete physical, mental, and social well-being, not merely the absence of disease or infirmity.” This multidimensional framework informs our operationalization through three measurement dimensions derived from the 2021 China General Social Survey (CGSS): physical health, mental health, and health-related functional limitations.

Physical health status assessment employed a dual-method approach: subjective self-evaluation using a 5-point Likert scale (1 = very unhealthy to 5 = very healthy), and objective classification via Body Mass Index (BMI) measurements (coded as 1 for normal range, 0 otherwise). Although BMI has limitations in reflecting health status, its inclusion provides a valid reference. Mental health was operationalized through self-reported frequency of depressive mood during the past 4 weeks using a 5-point scale (1 = always to 5 = never), while functional limitations were measured by reported interference frequency with work or daily activities on the same scale.

### 2.5 Mediating variables

Employment status serves as a mediating variable operationalized through three dimensions. First, employment participation distinguishes economically active individuals (coded 1 for currently employed) from inactive populations (coded 0 for non-employed). Second, occupational income uses logarithmically transformed labor earnings to address distributional skewness. Third, employment stability adopts contractual formalization as the criterion: formal employment with contracts (coded 1) versus informal arrangements without contractual protection (coded 0). This tripartite measurement captures workforce engagement intensity, economic returns, and institutional safeguards.

Medical expenditures constitute a critical mediating mechanism, measured through two complementary indicators. Healthcare utilization frequency adopts an ordinal scale with assigned values ranging from 0 for “never” seeking care, 1 for “approximately yearly” visits, 2 for “several times annually,” 3 for “monthly” consultations, 4 for “weekly” treatments, to 5 for “multiple times weekly” utilization, where higher values indicate the greater care-seeking frequency and presumed financial strain. Financial accessibility assesses cost-induced care rationing through the question “During the past 12 months, did you forgo necessary medical treatment due to cost?” with affirmative responses (coded 1) signaling expenditure-driven healthcare deprivation and negative responses (coded 0) reflecting adequate cost-bearing capacity.

### 2.6 Control variables

The study incorporates control variables—gender, age, urban–rural, and educational attainment—to account for potential confounding effects. Gender was dichotomized with males coded as 1 and females as 0. Age is calculated based on the respondent’s date of birth and is a continuous variable. Urban–rural was classified through household registration (hukou) status: non-agricultural hukou (including urban resident registration, and other non-farming categories) was coded as 1, while agricultural hukou was coded as 0. Educational attainment, recognized as a critical component of human capital alongside health, was quantified using formal schooling years. The coding scheme assigned 0 years to individuals with no formal education, 6 years to those completing literacy classes or elementary school, 9 years for junior high school graduates, 12 years for vocational/technical secondary education or regular high school completion, 15 years for junior college degrees (including both adult and conventional higher education), 16 years for bachelor’s degree holders, and 19 years for postgraduate qualifications. The descriptive statistics of all variables are presented in Table 1.

**Table 1:** Variable settings.

Variables	Observed variable	Min	Max	Mean/Ratio	SD
Relative poverty	Objective indicator	0	1	0.360	0.481
	Subjective indicator	0	1	0.090	0.279
Individual health	Physical health(subjective)	1	5	3.480	1.094
	Physical health(objective)	0	1	0.540	0.499
	Mental health	1	5	3.940	1.084
	Health-related functional limitations	1	5	3.940	1.230
Employment status	Employment participation,	0	1	0.450	0.498
	Occupational income	3.00	16.12	9.708	1.548
	Employment stability	0	1	0.530	0.499
Medical expenditures	Frequency of visits	0	5	1.320	1.114
	Affordability of medical expenditures	0	1	0.130	0.337
Control variable	Gender	0	1	0.450	0.498
	Age	18	99	51.610	17.565
	Urban–rural	0	1	0.410	0.491
	Education	0	19	9.340	4.677

## 3 Results

### 3.1 Descriptive statistics

Health serves as the foundation for individuals to engage in various activities and holds significant importance in human development. Benefiting from the advancement of China’s healthcare and public health initiatives, population health has shown substantial improvement.

As shown in Table 2, approximately 81.5% of respondents self-reported relatively good physical health status, while 89.3% demonstrated optimistic mental health conditions. However, the data reveals a prominent issue of obesity among residents, which aligns with the findings in recent editions of the “Report on Chinese Residents’ Nutrition and Chronic Disease Status” indicating the escalating obesity problem nationwide. Overall, health status exerts a significant influence on residents’ daily lives.

**Table 2:** The descriptive statistics of variables.

Variables	Observed variable	Frequency	Percentage (%)
Physical health (subjective)	Very unhealthy	428	5.3
	Somewhat unhealthy	1,069	13.2
	Average	2,283	28.1
	Somewhat healthy	2,855	35.1
	Very healthy	1,490	18.3
Physical health (objective)	BMI not within normal range	3,757	46.2
	BMI within normal range	4,368	53.8
Mental health	Always	199	2.4
	Often	669	8.2
	Sometimes	1814	22.3
	Rarely	2,150	26.5
	Never	3,293	40.5
Health-related functional limitations	Always	407	5
	Often	909	11.2
	Sometimes	1,232	15.2
	Rarely	1794	22.1
	Never	3,783	46.6
Relative poverty (objective)	Income above 50% of the median	5,171	63.6
	Income at or below 50% of the median	2,954	36.4
Relative poverty (subjective)	Not far below average	7,434	91.5
	Far below average	691	8.5

Against the backdrop of China’s historical eradication of absolute poverty, relative poverty has become increasingly prominent. According to Table 2 data, the objective relative poverty rate stands at 36.4%. However, only 8.5% of the population subjectively identifies as being in a state of “poverty.” This significant disparity between objective poverty lines and subjective poverty perception aligns with domestic and international research findings, profoundly revealing the limitations of relying solely on economic metrics to measure poverty (23). This divergence stems from the multidimensional and social nature of poverty itself. Once basic survival needs are met, individuals’ judgment of ‘poverty’ shifts toward social comparisons and perceptions of broader quality of life. The traditional concept of “not scarcity but inequality” coupled with heightened expectations for better lives amid rapid modernization further amplifies the subjective dimension of relative poverty. Therefore, in China’s new phase of advancing common prosperity and governing relative poverty, policy evaluation must transcend singular standards by incorporating subjective poverty assessments. This integration will form a comprehensive poverty measurement framework combining objective and subjective dimensions, enabling more precise identification of target populations, understanding of poverty drivers, and evaluation of policy effectiveness—ultimately serving the goal of enhancing the well-being of all citizens.

### 3.2 Model evaluation

Prior to model specification, normality testing was conducted on the data. All variables in this study exhibited skewness coefficients below 3 and kurtosis coefficients below 8, indicating that the skewness and kurtosis values of all observed variables met the prescribed criteria, thereby confirming the normality of the dataset. Following the model specification, identification procedures were performed. The preliminary model satisfied the t-rule, confirming the rationality of the model specification. Additionally, the analysis confirmed no occurrence of improper estimates (e.g., negative variances or out-of-range correlations), allowing progression to subsequent analyses.

The overall model fit was evaluated using established indices recommended by Marsh et al. (24), including the Root Mean Square Error of Approximation (RMSEA), Goodness-of-Fit Index (GFI), Adjusted Goodness-of-Fit Index (AGFI), Normed Fit Index (NFI), and Comparative Fit Index (CFI). Interpretation criteria were applied as follows: An RMSEA value below 0.05 indicates excellent model fit, while values between 0.05 and 0.08 suggest acceptable fit. GFI and AGFI values exceeding 0.9 demonstrate strong alignment between the model and empirical data, reflecting the model’s capacity to effectively explain observed patterns. Similarly, NFI and CFI values greater than 0.9 (with values approaching 1.0) signify superior model fit, indicating enhanced consistency between the hypothesized model and actual data.

During the evaluation phase, certain fit indices initially failed to meet these thresholds, necessitating model modification. Post-revision, all indices achieved the required standards, validating the final model’s statistical adequacy for substantive interpretation. The structure of the revised indicators is shown in Table 3.

**Table 3:** Table of goodness-of-fit indicators.

Indicator	Theoretical value	Actual value
RMSEA	<0.08	0.068
GFI	>0.9	0.957
AGFI	>0.9	0.930
NFI	>0.9	0.907
CFI	>0.9	0.909

### 3.3 Analysis of pathways

**Figure 2:**
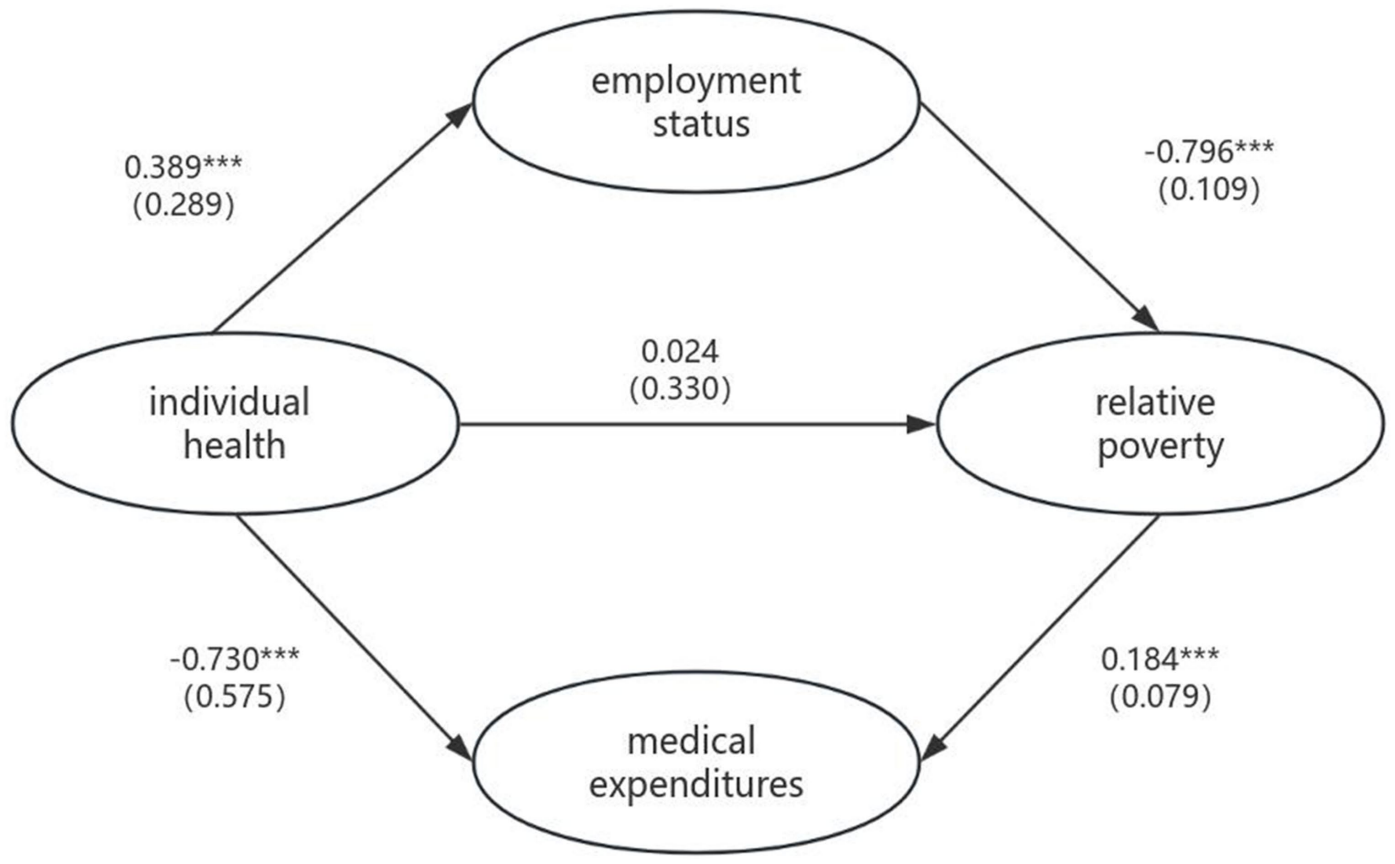
Path diagram of the relationship between latent variables. *p < 0.05, **p < 0.01, ***p < 0.001. () Represent standard errors.

Figure 2 presents the structural model and empirical findings regarding the mechanisms linking individual health to relative poverty.

This study constructed a preliminary conceptual model using structural equation modeling (SEM) based on extant literature. Following rigorous model refinement and adjustment, the final analytical framework was established, yielding statistically validated results with all model fit indices and parameter estimates demonstrating statistical significance. Key findings include:

#### 3.3.1 Observed poverty-alleviation effects associated with health

The analytical results indicate that the standardized total effect of individual health on relative poverty is −0.42, demonstrating a statistically significant negative correlation. According to Cohen’s criteria, these findings are categorized as a medium-to-large effect size, suggesting that health holds potential relevance in the dynamics of poverty. This provides preliminary empirical support for health-focused poverty alleviation strategies guided by health capital theory.

#### 3.3.2 Complete mediation by employment status and medical expenditures

Under covariate control, the magnitude (absolute value) of the standardized coefficient indicates the strength of the relationship between the variables and the sign indicates the direction of the relationship. Under the control of covariates, the standardized coefficient between health status and employment status was 0.389, while the standardized coefficient with relative poverty was −0.796. This indicates that improved health conditions enhance individuals’ income-generating capacity, thereby alleviating poverty. Simultaneously, the coefficient between health status and medical expenditure was −0.730, while medical expenditure showed a positive correlation coefficient of 0.184 with poverty. These findings demonstrate that health improvements can reduce medical expenditures and help prevent individuals from falling into relative poverty. The research results suggest that personal health status primarily affects relative poverty through employment status and medical expenditures.

#### 3.3.3 “Health-employment-poverty” constitutes the primary pathway

In the total effect of health on relative poverty, employment status accounts for approximately 69.75% of the mediating effect, highlighting that employment promotion represents a potential anti-poverty intervention. This implies that, compared to medical expenditures, health-related income loss may exert more persistent and systematic consequences. Notably, the standardized coefficient intensity of employment status directly affecting poverty incidence is significantly higher than other pathways, offering critical insights for leveraging employment-centered approaches in poverty governance.

### 3.4 Heterogeneity analysis

China’s urban–rural dual structure has generated profound social, economic, and cultural disparities between regions, resulting in disparate impacts on individual health and poverty vulnerability. Urban areas, as socioeconomic centers, typically offer more comprehensive healthcare systems and higher levels of health protection. Conversely, rural areas face systemic disadvantages due to resource constraints and inadequate infrastructure. This structural imbalance heightens rural residents’ exposure to health risks and increases their likelihood of health-induced poverty. Simultaneously, the occupational characteristics of rural residents—primarily involving high-physical-intensity labor—intensify their dependence on physical health, establishing it as a fundamental determinant of livelihood sustainability.

**Figure 3:**
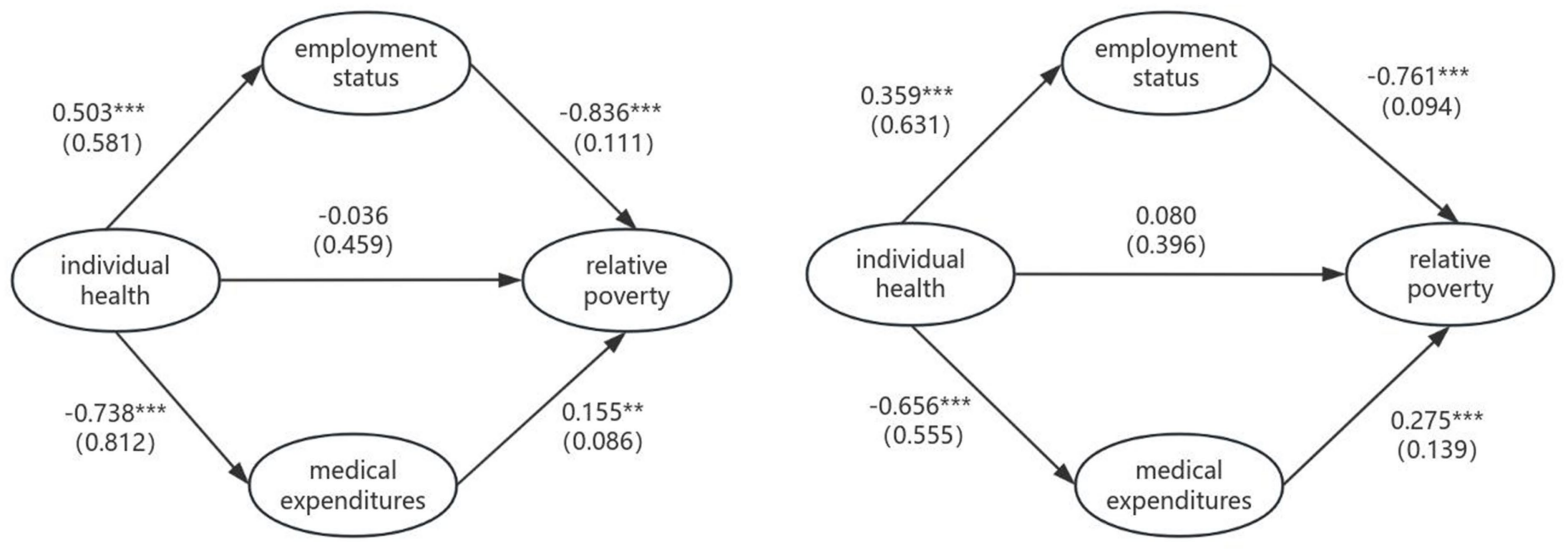
Pathway maps for rural clusters (left) and urban clusters (right). *p < 0.05, **p < 0.01, ***p < 0.001. () Represent standard errors.

Consequently, this study investigates how personal health mediates urban–rural differentials in relative poverty vulnerability, with empirical results systematically presented in Figure 3. The standardized total effect of health-driven poverty reduction among rural respondents was −0.571, compared to −0.374 for urban respondents. This indicates that individuals with agricultural hukou status achieve superior poverty alleviation outcomes through health improvements. This disparity primarily stems from their constrained capabilities due to economic and social limitations. Health enhancement substantially boosts their labor productivity and productive efficiency, thereby strengthening economic agency and social participation capacity. Conversely, although urban hukou holders also benefit from health improvements, their preexisting higher capability endowments result in diminished marginal returns of health on poverty reduction.

## 4 Discussion

Based on the 2021 China General Social Survey data, this study examines the impact of individual health on relative poverty—a critical research area in China’s poverty governance during the post-poverty era. The findings demonstrate that health serves a significant poverty reduction function: improved health conditions contribute to enhancing human capital while mitigating medical expenditure, thereby reducing or preventing the risk of relative poverty. Although China eradicated absolute poverty in 2020, health-induced poverty remains a persistent contributing factor (4, 6, 25, 26). Furthermore, compounded by the strain exerted by the COVID-19 pandemic on public health systems (4), prioritizing health considerations in poverty governance has become an urgent priority for consolidating poverty reduction gains and preventing relapse into poverty (21).

A key finding of this study is that individual health primarily influences relative poverty through employment status and medical expenditures. This pathway mechanism aligns with established empirical patterns. Simultaneously, the research validates theoretical propositions from human capital theory and poverty trap theory regarding health-poverty dynamics. Within the human capital framework, health constitutes fundamental capital for maintaining labor capacity; health deterioration directly undermines income-generating ability by reducing employment quality and working hours (3, 11, 12), thereby increasing poverty vulnerability. Concurrently, the poverty trap mechanism demonstrates how health shocks trigger catastrophic medical expenditures, creating “health-induced poverty” (17). Consequently, health-centered poverty interventions must not only address health per se but also incorporate employment enhancement and medical expenditure reduction to achieve targeted interventions.

This study further elucidates the “Health-Employment-Poverty” transmission mechanism. The findings demonstrate that health-related income loss generally exerts a greater influence in leading to poverty than medical expenditures (10). Health constitutes the foundation of human capital. When health deteriorates, an individual’s labor capacity becomes severely compromised, resulting in reduced income or unemployment. Such health-induced income loss not only directly lowers living standards but also generates multifaceted negative effects, thereby exacerbating relative poverty (25, 27). In contrast, while medical expenditure remains a key factor causing poverty, its impact is largely confined to direct economic outlays. Although high medical costs impose economic pressure, this burden may gradually lessen as health improves. This discovery challenges the traditional poverty theory’s over-reliance on income subsidies and substantiates Sen’s Capability Approach framework (9, 28). An alternative explanation merits attention: the continuous advancement of China’s public health system in recent years, coupled with the expansion of healthcare coverage, has alleviated the economic shocks associated with illness (8, 29), consequently mitigating poverty caused by medical expenditures. Although the precise mechanisms require further empirical validation, our findings suggest that the employment pathway deserves prioritization in the design of health-related poverty reduction policies.

Utilizing nationally representative data, this study analyzes the relationship between individual health and relative poverty status, examines its underlying mechanisms, and conducts an in-depth exploration incorporating China’s urban–rural heterogeneity. It thereby provides empirical evidence from the Chinese context for the literature on health-centered poverty alleviation. Building on these findings, this paper further proposes policy recommendations for designing poverty reduction interventions from the perspective of enhancing individual health. This approach not only aligns with China’s strategic demands in the post-poverty-eradication era but also advances the guidance of the “Healthy China” initiative. Thus, policymakers should give full consideration to the poverty reduction potential inherent in health factors within future anti-poverty governance systems.

## 5 Conclusion

This empirical study examines the impact of individual health on relative poverty and systematically analyzes its underlying mechanisms. Furthermore, it explores the significant role of health improvement within poverty alleviation strategies. Based on these findings, this study contends that China’s anti-poverty governance should prioritize the following dimensions. First, strengthen public health infrastructure to establish an integrated “prevention-treatment-rehabilitation” system. Second, create higher-quality employment opportunities while formulating flexible labor policies. Concurrently, optimize the basic medical insurance system and implement multi-tiered supplementary insurance schemes to mitigate poverty risks induced by medical expenditures. Additionally, considering urban–rural regional disparities, spatial inequalities should be addressed through progressively balanced allocation of healthcare resources—particularly by enhancing service accessibility in underserved areas. These comprehensive measures constitute a holistic governance model that disrupts the health-poverty trap through dual employment-medical expenditure channels, thereby advancing health equity as a cornerstone of sustainable social development.

However, this study has four noteworthy limitations. First, constrained by data availability, this cross-sectional analysis cannot establish temporal causal relationships. Should updated national survey datasets including key variables be established in the future, further research using recent data should be conducted for assessment. Second, the structural equation modeling framework assumes linear relationships, whereas empirical patterns suggest potential nonlinear dynamic relationships between health and poverty. Third, although operationalizing relative poverty through income-based indicators offers methodological convenience, it overlooks disparities in wealth accumulation and asset-based deprivation. Future research could integrate wealth indices with income metrics to enhance conceptual rigor. Fourth, the analysis focuses exclusively on the link between health and relative poverty, without exploring comparative mechanisms between health and absolute poverty—a topic of significant importance.

## Data Availability

The raw data are publicly available in the China National Survey Data Archive (http://www.cnsda.org/). Processed datasets derived from data cleaning and structuring are available from the corresponding author upon request.
